# Differential Tropism of SARS-CoV and SARS-CoV-2 in Bat Cells

**DOI:** 10.3201/eid2612.202308

**Published:** 2020-12

**Authors:** Susanna K.P. Lau, Antonio C.P. Wong, Hayes K.H. Luk, Kenneth S.M. Li, Joshua Fung, Zirong He, Flora K.K. Cheng, Tony T.Y. Chan, Stella Chu, Kam Leng Aw-Yong, Terrence C.K. Lau, Kitty S.C. Fung, Patrick C.Y. Woo

**Affiliations:** The University of Hong Kong, Hong Kong, China (S.K.P. Lau, A.C.P. Wong, H.K.K. Luk, K.S.M. Li, J. Fung, Z. He, F.K.K. Cheng, T.T.Y. Chan, S. Chu, K.L. Aw-Yong, P.C.Y. Woo);; City University of Hong Kong, Hong Kong (T.C.K. Lau);; United Christian Hospital, Kwun Tong, Hong Kong (K.S.C. Fung)

**Keywords:** coronavirus disease, SARS-CoV-2, severe acute respiratory syndrome coronavirus 2, severe acute respiratory syndrome, SARS, viruses, respiratory infections, zoonoses, COVID-19, SARS-related coronavirus, bat, tropism, origin

## Abstract

Severe acute respiratory syndrome coronavirus 2 did not replicate efficiently in 13 bat cell lines, whereas severe acute respiratory syndrome coronavirus replicated efficiently in kidney cells of its ancestral host, the *Rhinolophus sinicus* bat, suggesting different evolutionary origins. Structural modeling showed that RBD/*Rs*ACE2 binding may contribute to the differential cellular tropism.

Coronavirus disease (COVID-19) is a global pandemic, affecting 213 countries with >2.7 million confirmed cases and 190,000 fatalities as of April 25, 2020 (*1*). Its causative agent was identified as severe acute respiratory syndrome coronavirus (SARS-CoV) 2 (SARS-CoV-2), which belongs to the same coronavirus species as SARS-CoV and SARS-related CoVs (SARSr-CoVs) in horseshoe bats (genus *Rhinolophus*) (*2*,*3*). Given the history among some early case-patients of visiting the Huanan seafood market in Wuhan, China, and its genetic close relatedness to SARSr-CoVs in bats and pangolins (*2*,*4*), SARS-CoV-2 was suspected to have emerged from wild animals, particularly bats, similar to SARS-CoV. SARS-CoV was a recombinant virus that originated from Chinese horseshoe bats (*Rhinolophus sinicus*) before it infected palm civets and then humans (*5*). 

Studying cellular tropism may provide clues to the host range and possible origin of zoonotic viruses. For example, SARS-CoV could replicate efficiently in kidney cells of its primary origin, *R*. *sinicus*, but not in other tested bat cells (*6*). To elucidate the possible origin of SARS-CoV-2, we tested susceptibilities of bat cell lines developed from different species commonly found in southern China to infection by SARS-CoV-2 in comparison with SARS-CoV. The selected bat species harbored a diverse set of coronaviruses, including SARSr-CoVs and Middle East respiratory syndrome–related coronaviruses (MERSr-CoVs), which pose potential health threats to humans (*7*). We also performed structural modeling of the virus/host receptor-binding interface.

## The Study

SARS-CoV strain HKU-39849 was isolated in Hong Kong during the SARS epidemic as previously described (*8*). SARS-CoV-2 strain HK20 was isolated from a patient with COVID-19 in Hong Kong in early February 2020 (*3*). Thirteen primary or immortalized bat cell lines from 6 different bat species were subjected to infection with SARS-CoV and SARS-CoV-2 at multiplicity of infection of 0.1 as described previously (*6*,*9*,*10*), except with the addition of 2 µg/mL trypsin. The bat species included *Miniopterus pusillus*, *Pipistrellus abramus* (harboring *Pipistrellus*-BatCoV-HKU5), *R. sinicus* (harboring SARSr-BatCoVs, *Rhinolophus*-BatCoV-HKU2, *Rhinolophus sinicus*-BatCoV-HKU32), *Tylonycteris pachypus* (harboring *Tylonycteris*-BatCoV-HKU4), *Rousettus leschenaultii* (harboring many viruses, including *Rousettus*-BatCoV-HKU9 and *Rousettus*-BatCoV-HKU10), and *Myotis ricketii* (harboring *Myotis*-BatCoV-HKU6). Vero cells from African green monkey kidney were used as positive control (Appendix). We determined viral replication efficiency by quantitative reverse transcription PCR (qRT-PCR) on cell culture supernatants (Table 1) (*6*). Cells were considered susceptible to viral infection if qRT-PCR on day 5 postinfection showed >1 log_10_ increase in viral titer with statistical significance (p<0.05 by Student *t*-test).

**Table 1:** Primers used for reverse transcription quantitative PCR in study of coronavirus in bats*

Target	Primers, 5¢ ® 3¢		
	Forward	Reverse	Probe
SARS-CoV N gene CDC_N3	GGGAGCCTTGAATACACCAAAA	TGTAGCACGATTGCAGCATTG	(FAM) AYCACATTGGCACCCGCAATCCTG (BHQ1)
β-actin	CTCTTCCAGCCCTCCTTCCT (for bat cells) or CTCTTCCAGCCTTCCTTCCT (for human cells)	TTCATCGTGCTGGGAGCC (for bat cells) or TTCATTGTGCTGGGTGCC (for human cells)	(FAM) CATGAAGTGYGACGTBGACATCCG(BHQ1)

*CoV, coronavirus; N, nucleocapsid protein; SARS, severe acute respiratory syndrome.

**Figure 1:**
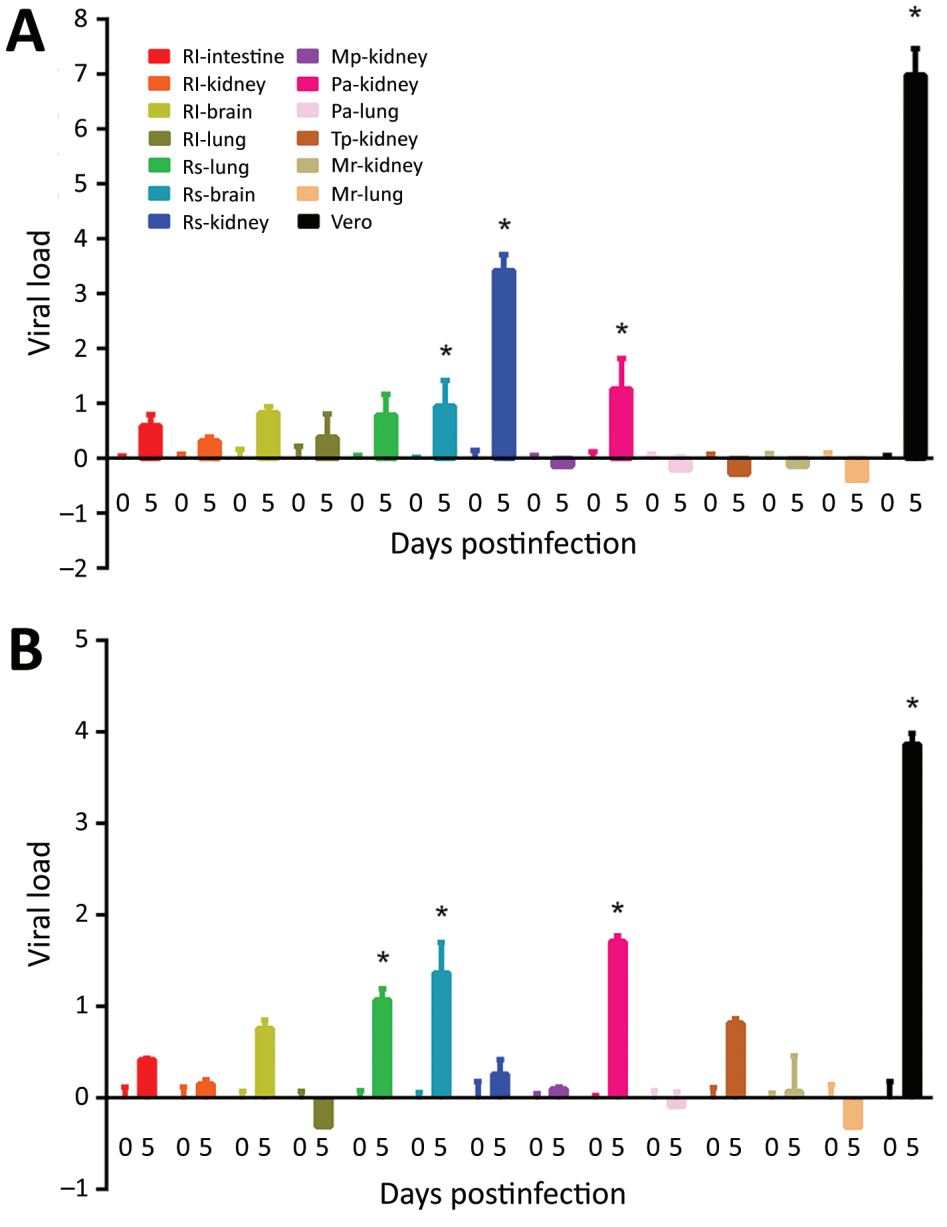
Susceptibilities of 13 bat cell lines to infection by SARS-CoV (A) and SARS-CoV-2 (B) shown from harvest of supernatants and cell lysates at day 0 and 5 postinfection. Viral titers and β-Actin mRNA were determined by real-time quantitative reverse transcription PCR. Viral load is expressed as normalized fold change in log_10_. Error bars indicate SDs of triplicate samples. Bat cell lines are listed by species and organ. Vero cells served as controls. Asterisk (*) indicates p<0.05 and increase in viral load >1 log_10_. Mp, *Miniopterus pusillus*, Mr, *Myotis ricketti;* Pa, *Pipistrellus abramus*, Rl, *Rousettus leschenaultii*, Rs, *Rhinolophus sinicus*, Tp, *Tylonycteris pachypus.* SARS-CoV, severe acute respiratory syndrome coronavirus.

SARS-CoV but not SARS-CoV-2 can replicate efficiently in *R. sinicus* kidney cells; SARS-CoV showed 3.48 log_10_-fold increase in viral titer. In contrast, only SARS-CoV-2 can replicate in *R. sinicus* lung cells, but at a low viral titer (1.08 log_10_-fold increase). Moreover, SARS-CoV-2 can replicate more efficiently (1.46 log_10_-fold increase) in *R. sinicus* brain cells than SARS-CoV (1.09 log_10_-fold increase), albeit still at low viral titer (Table 2; Figure 1). Both SARS-CoV and SARS-CoV-2 can also replicate in *P. abramus* kidney cells with low viral titers: 1.45 log_10_-fold increase for SARS-CoV and 1.71 log_10_-fold increase for SARS-CoV-2. We observed cytopathic effects in SARS-CoV–infected *R. sinicus* kidney cells and SARS-CoV– or SARS-CoV-2–infected *P. abramus* kidney cells with rounding of cells (Appendix). We performed immunofluorescence assay on those cell lines with >1 log_10_-fold increase in viral load (Appendix). *M. pusillus* kidney cells; *R. leschenaultii* kidney, brain, intestine, and lung cells; *T. pachypus* kidney cells; and *M. ricketii* kidney and lung cells did not support SARS-CoV or SARS-CoV-2 infection. Furthermore, both SARS-CoV and SARS-CoV-2 replicated less efficiently in Vero cells at 33°C than at 37°C, whereas no difference in viral replication in *R. sinicus* kidney cells was observed between 33°C and 37°C (Appendix).

**Table 2:** Viral load changes and cytopathic effects of severe acute respiratory syndrome coronavirus and coronavirus 2 in different cell lines on day 5 postinfection*

Cell lines	SARS-CoV				SARS-CoV-2		
	Viral load change, log_10_	p value	CPE		Viral load change, log_10_	p value	CPE
*Rousettus leschenaultii* intestine	0.63	0.0083	–		0.59	0.0039	–
*Rousettus leschenaultii* kidney	0.33	0.0071	–		0.15	0.0950	–
*Rousettus leschenaultii* brain	0.84	0.0019	–		0.77	0.0004	–
*Rousettus leschenaultii* lung	0.39	0.2345	–		−0.31	0.1224	–
*Rhinolophus sinicus* lung	0.91	0.0226	–		1.08	0.0002	–
*Rhinolophus sinicus* brain	1.09	0.0251	–		1.46	0.0022	–
*Rhinolophus sinicus* kidney	3.48	<0.0001	+		0.28	0.1280	–
*Miniopterus pusillus* kidney	−0.14	0.0372	–		0.10	0.0241	–
*Pipistrellus abramus* kidney	1.45	0.0176	+		1.71	<0.0001	+
*Pipistrellus abramus* lung	−0.21	0.2401	–		−0.09	0.4218	–
*Tylonycteris pachypus* kidney	−0.27	0.0051	–		0.82	0.0003	–
*Myotis ricketti* kidney	−0.14	0.1683	–		0.07	0.7615	–
*Myotis ricketti* lung	−0.41	0.0289	–		−0.32	0.0240	–
Vero	7.12	<0.0001	+		3.88	<0.0001	+

*CoV, coronavirus; CPE, cytopathic effects; SARS, severe acute respiratory syndrome.

**Figure 2:**
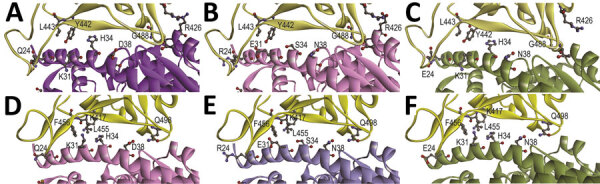
Structural modeling of the human (A, D), *Rhinolophus sinicus* bat (Rs-bat) (B, E), and *Pipistrellus abramus* bat (Pa-bat) (C, F) ACE2 with the receptor-binding domain (RBD) of the spike proteins of SARS-CoV and SARS-CoV-2. The models of RBDs of SARS-CoV and SARS-CoV-2 (yellow) are shown with human (purple), Rs-bat (pink). and Pa-bat (green) ACE2 structures in ribbon diagrams. The interface of different RBDs and human/bat ACE2 are shown and the residues with potential impact on binding affinity are shown in ball-and-stick format. Images were produced using Discovery Studio visualizer (Accelrys, https://www.accelrys.com).

To elucidate whether the receptor-binding interface is a contributing factor for cellular tropism, we modeled the structure of the SARS-CoV-2 receptor binding domain (RBD) with that of human angiotensin-converting enzyme 2 (hACE2), *R. sinicus* angiotensin-converting enzyme 2 (Rs-ACE2), and *P. abramus* angiotensin-converting enzyme 2 (Pa-ACE2) using homology modeling by SWISS-MODEL (https://swissmodel.expasy.org) as described previously (*11*), based on the crystal structure of SARS-CoV-RBD/hACE2. The sequence identity between SARS-CoV RBD (template) and SARS-CoV-2 RBD (template) was >50% and the interface for all RBD/ACE2 was similar (Figure 2). We identified 11 aa differences between SARS-CoV RBD and SARS-CoV-2 RBD sequences that involved 4 of 5 critical residues for hACE2 binding in SARS-CoV RBD. Y442 was one of the 5 critical residues in SARS-CoV RBD. Because F456 is more hydrophobic than Y442 in SARS-CoV-2 RBD, it may disturb the electrostatic interaction with hACE2/Rs-ACE2. The interface for RBD/Pa-ACE 2 was similar to that of RBD/hACE2 (Figure 2), implying that Pa-ACE2 may also serve as the host receptor for SARS-CoV and SARS-CoV-2.

## Conclusions

The ability of SARS-CoV but not SARS-CoV-2 to replicate in *R. sinicus* kidney cells, consistent with previous findings (*12*), may suggest a different evolutionary origin and path of SARS-CoV-2. SARS-CoV was most closely related to SARSr-*Rs*-BatCoVs from Yunnan, China, suggesting *R. sinicus* as its primary origin. It could also use *Rs*-ACE2 as receptor for cell entry (*13*), which may explain the efficient replication of SARS-CoV in *R. sinicus* kidney cells. Although SARS-CoV-2 is closely related to SARSr-CoVs in bats and pangolins, none of the existing animal viruses represents the immediate ancestor of SARS-CoV-2. SARS-CoV-2 was most closely related to SARSr-*Ra*-BatCoV-RaTG13 (96.1% genome identity) in *Rhinolophus affinis* from Pu’er, Yunnan (*2*), except that its RBD region was closest to pangolin-SARSr-CoV-MP789 (86.9% nucleotide identity) in smuggled pangolins from Guangdong, suggesting that SARS-CoV-2 may have evolved through recombination (*3*). The inability of SARS-CoV-2 to efficiently infect and replicate in *R. sinicus* cells may imply that *R. sinicus* bats were unlikely to be its proximal origin. However, bats are the primary origin of SARS-CoV, human coronavirus 229E (HCoV-229E), and probably MERS-CoV; therefore, SARS-CoV-2 most likely originated from bats. One possibility is that SARS-CoV-2 has restricted bat species tropism. Other bat species, such as *R. affinis*, may harbor the ancestor of SARS-CoV-2 and can be tested for cellular susceptibilities in future studies. It is also possible that SARS-CoV-2 can no longer replicate in bat cells because of substantial genetic adaptation, such as through natural evolution in an intermediate host before infecting humans.

The difference in critical residues for receptor binding between SARS-CoV and SARS-CoV-2 may have contributed to their differential infectivities in *R. sinicus* cells, as suggested by results from structural modeling of the receptor-binding interface. Whereas SARS-CoV RBD was most closely related to SARSr-*Rs*-BatCoV-WIV1 from *R. sinicus*, SARS-CoV-2 RBD was most closely related to the RBD region of pangolin-SARSr-CoV-MP789 from pangolins (*14*). Mutagenesis studies are needed to investigate whether changes of these amino acid sites may affect binding affinity to the ACE2 of different hosts and restore the infectivity of SARS-CoV-2 in *R. sinicus* cells.

The restricted cellular tropism of SARS-CoV and SARS-CoV-2 is different from that of MERS-CoV, which showed broad species tropism in bat cells. MERS-CoV could replicate in >5 bat cell lines (*M. ricketti* lung, *P. abramus* kidney, *R. sinicus* kidney and lung, and *R. leschenaultii* kidney cells) from 3 bat families (*6*). Although dromedary camels were the immediate source of MERS-CoV, bats were suggested to be the ultimate evolutionary origin (*10*,*15*). Of note, SARS-CoV, SARS-CoV-2, and MERS-CoV could all replicate in *P. abramus* kidneys at low titers. Structural modeling supported that *P. abramus* ACE2 could serve as host receptor for SARS-CoV and SARS-CoV-2. *P. abramus* is known to harbor *Pi*-BatCoV-HKU5 from the subgenus *Merbecovirus* (containing MERS-CoV) but not members of *Sarbecovirus* (containing SARS-CoV and SARS-CoV-2) (*10*,*15*). *P. abramus* is a potential accidental host for spillover of and source for emergence of diverse coronaviruses including SARSr-CoVs.

Appendix Additional information about tropism of SARS coronavirus and SARS coronavirus 2 in bat cells.
