# Supplementary material for: Differential Tropism of SARS-CoV and SARS-CoV-2 in Bat Cells
Source: Emerg Infect Dis. 2020 Dec;26(12):2961–5. doi: 10.3201/eid2612.202308 (PMC7706959; doi:10.3201/eid2612.202308)
Supplement: Appendix — Additional information about tropism of SARS coronavirus and SARS coronavirus 2 in bat cells. [file 20-2308-Techapp-s1.pdf]

Article DOI: https://doi.org/10.3201/eid2612.202308

Differential Tropism of SARS-CoV and SARS-CoV-2 in Bat Cells

Appendix

We tested 13 cell lines from *Rousettus leschenaultii* bat (intestine, kidney, brain, lung), *Rhinolophus sinicus* bat (lung, brain, kidney), *Miniopterus pusillus* bat (kidney), *Pipistrellus abramus* bat (kidney, lung), *Tylonycteris pachypus* bat (kidney), and *Myotis ricketti* bat (kidney, lung). Cells were subject to infection by SARS-CoV and SARS-CoV-2 viruses (isolated from Vero cells, Passage 6) with MOI of 0.1 and with 2 post-adsorption washings. We performed the testing in triplicate. Supernatants and cell lysates were harvested at day 0 and day 5 postinfection. Viral titers and β-Actin mRNA were determined by real-time quantitative RT-PCR. Viral load was expressed as normalized fold change in $\log_{10}$. *Rhinolophus sinicus* brain and kidney cells and *Pipistrellus abramus* kidney cells can support SARS-CoV infection, demonstrated by $\geq$1 $\log_{10}$ increase in viral load at day 5 ($p<0.05$). *Rhinolophus sinicus* lung and brain cells and *Pipistrellus abramus* kidney cells support SARS-CoV-2 infection.

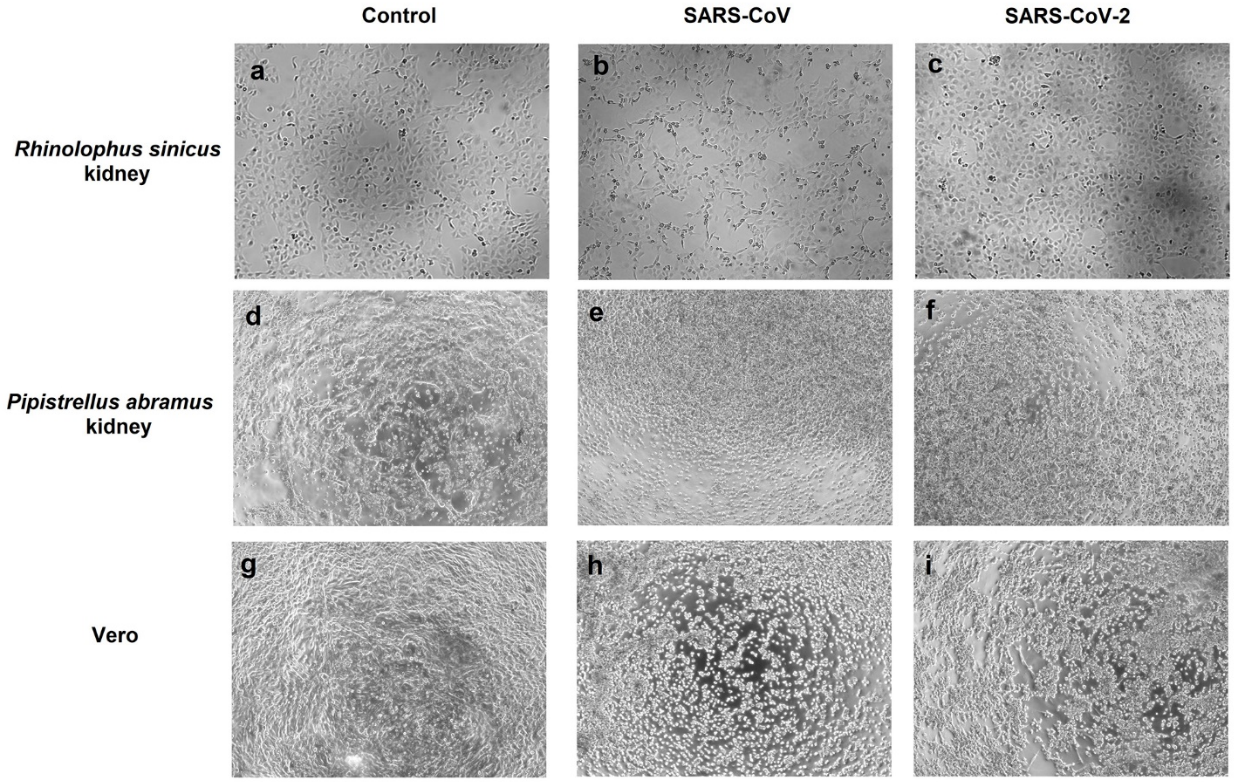

Appendix Figure 1. Cytopathic effects (CPE) in infected *Rhinolophus sinicus* kidney, *Pipistrellus abramus* kidney and Vero cells on day 5 postinfection. CPE was compared between *Rhinolophus sinicus* kidney cells that were uninfected (control) (A), and infected with SARS-CoV (B) and SARS-CoV-2 (C). CPE was compared between *P. abramus* kidney cells that were uninfected (control) (D), infected with SARS-CoV (E) and SARS-CoV-2 (F). CPE was compared between Vero cells that were uninfected (control) (G), infected with SARS-CoV (H) and SARS-CoV-2 (I).

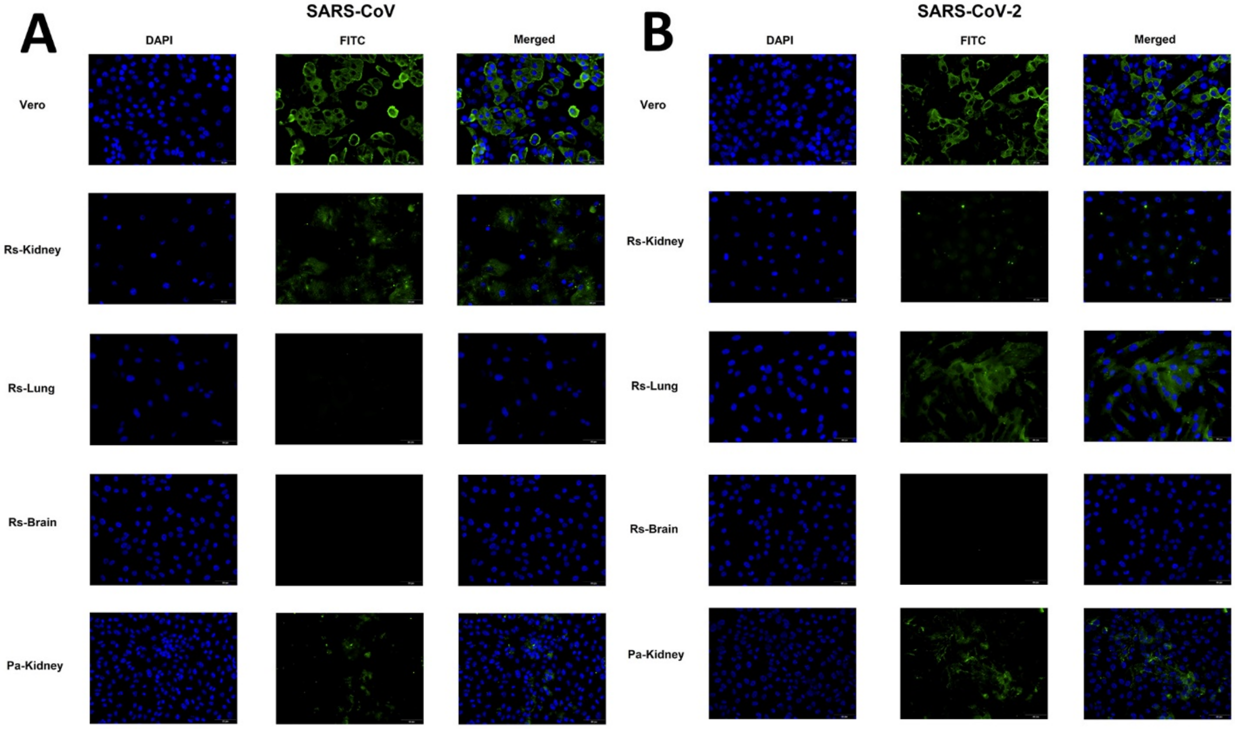

Appendix Figure 2. Antigen expression of SARS-CoV and SARS-CoV-2 in Vero and bat cell lines. Selected cell lines were subject to infection by SARS-CoV (A) and SARS-CoV-2 (B). Antigen expression of SARS-CoV and SARS-CoV-2 with MOI of 0.1 were assessed with immunofluorescence staining. Infected cells were fixed with 4% paraformaldehyde at 24 hpi and immunolabelled with rabbit anti-SARS-CoV-NP and SARS-CoV-2-NP primary antibody, followed by fluorescein isothiocyanate (FITC) conjugated goat-antirabbit antibody and mounting solution with DAPI stain. Bars represent 50 μm.

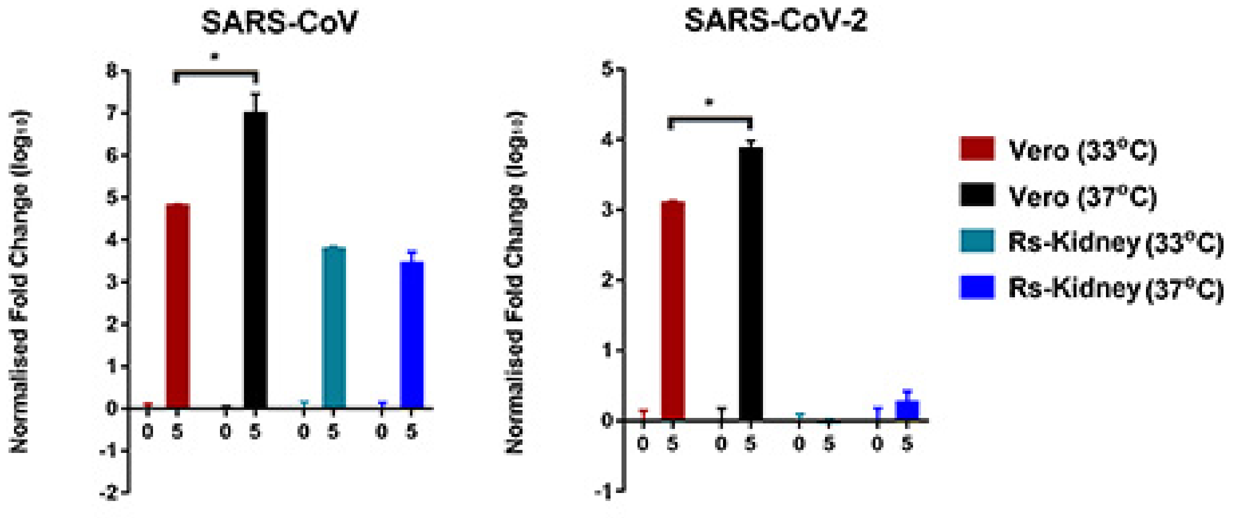

Appendix Figure 3. Susceptibilities of Vero and *Rhinolophus sinicus* kidney cell lines to infection by SARS-CoV and SARS-CoV-2 at different temperatures. *Rhinolophus sinicus* kidney (Rs-Kidney) and Vero cells were subject to infection by SARS-CoV (A) and SARS-CoV-2 (B) with MOI of 0.1 at 33°C and 37°C. Supernatants and cell lysates were harvested at day 0 and day 5 postinfection. Viral titers and β-Actin mRNA were determined by real-time quantitative RT-PCR. Viral load was expressed as normalized fold change in log_{10}. Standard deviations of triplicate samples were shown. Asterisk * indicates $p<0.05$.
